# Supplementary material for: Application of a Loop-Mediated Isothermal Amplification (LAMP) Assay for the Detection of Listeria monocytogenes in Cooked Ham
Source: Foods. 2023 Jan 1;12(1):193. doi: 10.3390/foods12010193 (PMC9818245; doi:10.3390/foods12010193)
Supplement: Supplementary file 1 [file foods-12-00193-s001.zip › Figure S1 Legend.pdf]

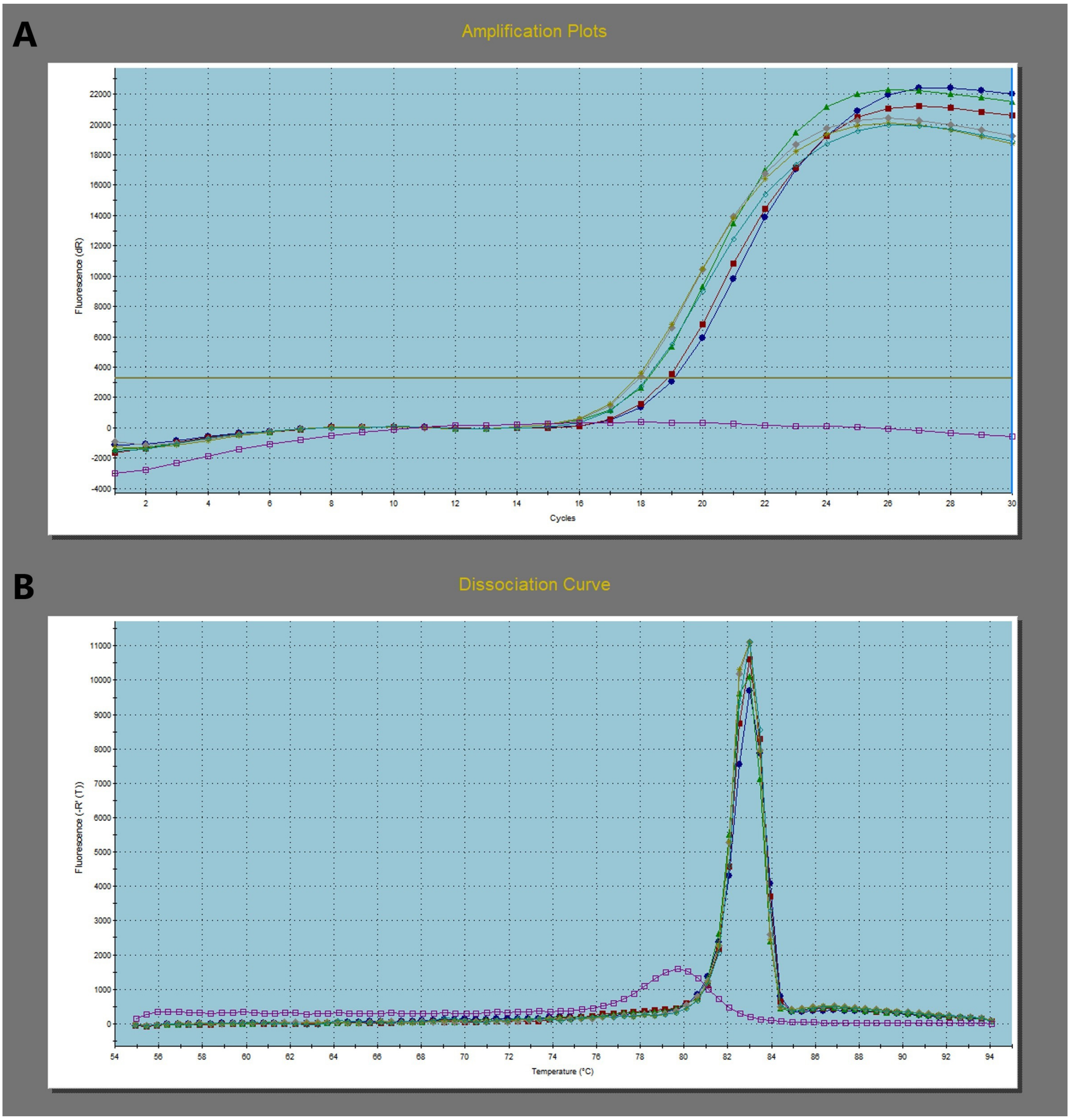

Figure S1. Specificity of Real-Time LAMP PCR assay using DNA isolated from *L. moncytogenes* strains; (A) amplification curves, (B) melting temperature. *L. monocytogenes* ATCC 13932 blue lane, *L. monocytogenes* ATCC 35152 red lane, *L. monocytogenes* ATCC 7644 green lane, *L. monocytogenes* ATCC 19111 grey lane, *L. monocytogenes* ATCC 19115 Golden yellow lane, Positive control light blue lane, Negative control fuchsia lane.
