# Supplementary material for: Application of a Loop-Mediated Isothermal Amplification (LAMP) Assay for the Detection of Listeria monocytogenes in Cooked Ham
Source: Foods. 2023 Jan 1;12(1):193. doi: 10.3390/foods12010193 (PMC9818245; doi:10.3390/foods12010193)
Supplement: Supplementary file 1 [file foods-12-00193-s001.zip › Figure S2 Legend.pdf]

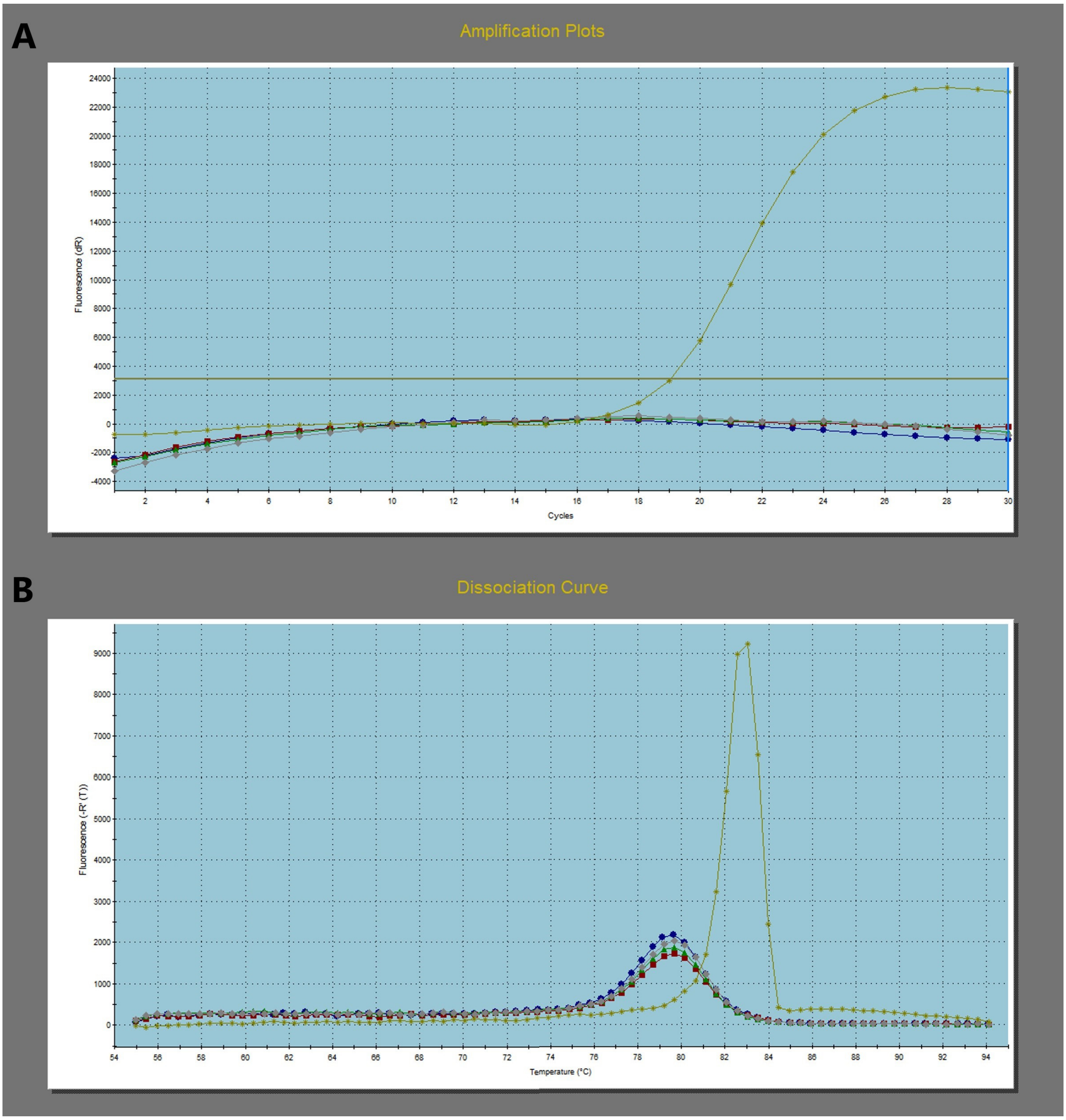

Figure S6. Specificity of Real-Time LAMP PCR assay using DNA isolated from non-*L. monocytogenes* strains; (A) amplification curves, (B) melting temperature; *Listeria innocua* ATCC 33090 grey lane, *Listeria ivanovii* ATCC 19119 red lane, *Listeria seeligeri* CLISS 11 blue lane, Positive control golden yellow lane, Negative control green lane.
