# Supplementary material for: Application of a Loop-Mediated Isothermal Amplification (LAMP) Assay for the Detection of Listeria monocytogenes in Cooked Ham
Source: Foods. 2023 Jan 1;12(1):193. doi: 10.3390/foods12010193 (PMC9818245; doi:10.3390/foods12010193)
Supplement: Supplementary file 1 [file foods-12-00193-s001.zip › Figure S3 Legend.pdf]

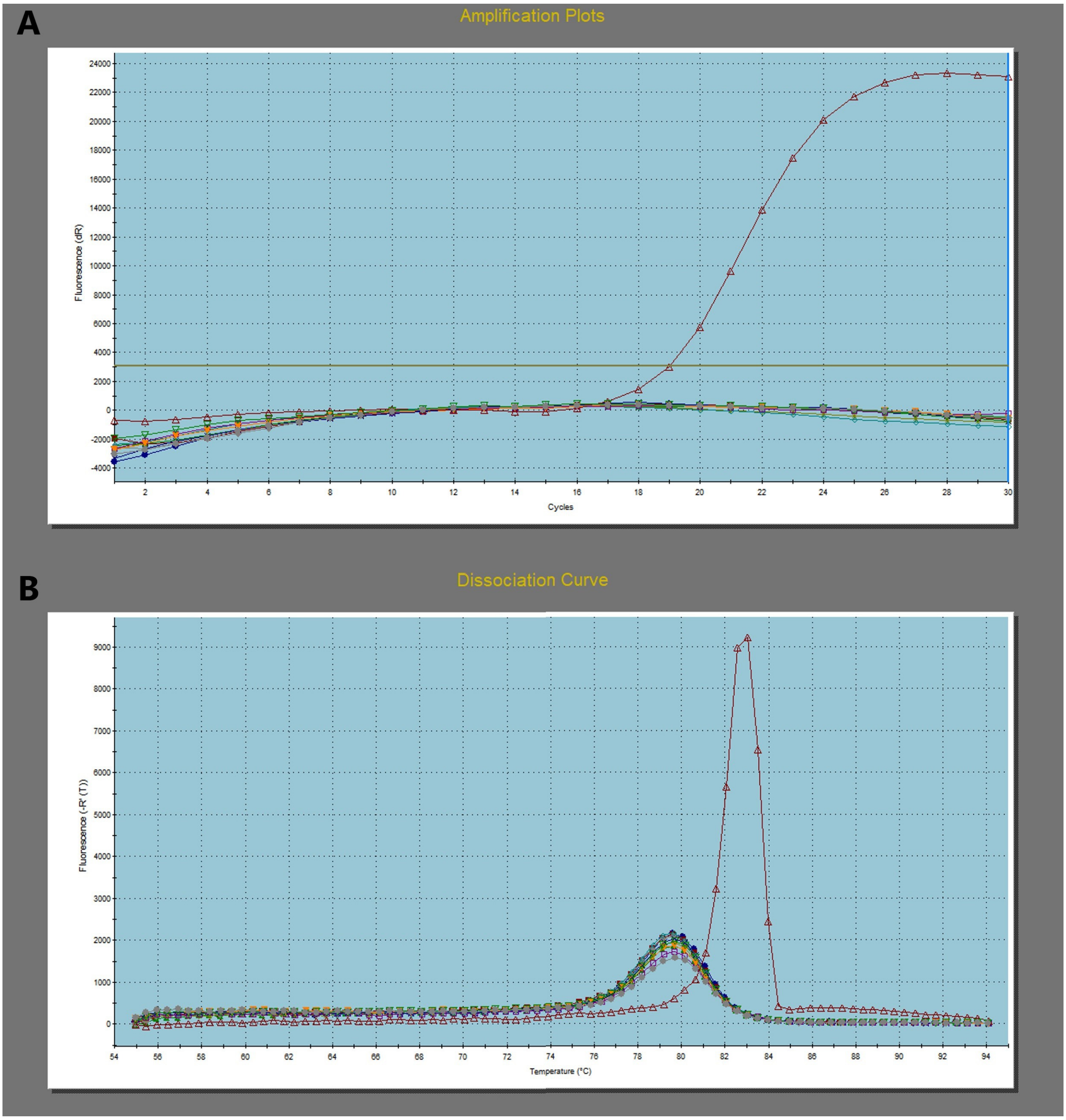

Figure S7. Specificity of Real-Time LAMP PCR assay using DNA isolated from other strains; (A) amplification curves, (B) melting temperature; *Escherichia coli* ATCC 25922 green line, *Salmonella Enteritidis* ATCC 13076 orange lane, *Salmonella Typhimurium* ATCC 14928 fuchsia lane, *Staphylococcus aureus* ATCC 25923 light blue, *Enterococcus faecalis* ATCC 29212 golden yellow lane, *Enterobacter aerogenes* ATCC 13048 blue lane, *Pseudomonas aeruginosa* ATCC 9027 dark red lane, *Bacillus cereus* ATCC 6633 light grey lane, *Citrobacter freundii* ATCC 8090 yellow lane, *Proteus vulgaris* ATCC 13315 dark green lane, Positive control red lane, Negative control grey lane.
